# Expression of Musashi-1 During Osteogenic Differentiation of Oral MSC: An In Vitro Study

**DOI:** 10.3390/ijms20092171

**Published:** 2019-05-02

**Authors:** Miguel Padial-Molina, Juan G. de Buitrago, Raquel Sainz-Urruela, Dario Abril-Garcia, Per Anderson, Francisco O’Valle, Pablo Galindo-Moreno

**Affiliations:** 1 Department of Oral Surgery and Implant Dentistry. School of Dentistry, University of Granada, Granada 18071, Spain; mipadial@ugr.es (M.P.-M.); je.gonzalezdebuitrago@gmail.com (J.G.d.B.); raquelsainz@ugr.es (R.S.-U.); darioabril@ugr.es (D.A.-G.); 2 Servicio de Analisis Clinicos e Inmunologia, UGC Laboratorio Clinico, Hospital Universitario Virgen de las Nieves, Granada 18014, Spain; per.anderson@genyo.es; 3 Biosanitary Institute of Granada (ibs.Granada). University of Granada, Granada 18071, Spain; fovalle@ugr.es; 4 Department of Pathology and IBIMER. School of Medicine, University of Granada, Granada 18071, Spain

**Keywords:** musashi-1, periostin, *RUNX2*, mesenchymal stromal cells, osteogenic differentiation, bone regeneration, bone healing

## Abstract

Background: Musashi-1 (MSI1) is a negative regulator of mesenchymal stromal cell (MSC) differentiation which in turn favors cell proliferation. However, little is known about its expression by MSC from the oral cavity and in the context of osteogenic differentiation. Aim: The aim of this study was to analyze the expression of MSI1 in the context of osteogenic differentiation of MSC derived from the oral cavity. Material/methods: For this in vitro study, MSC were isolated from six different origins of the oral cavity. They were extensively characterized in terms of proliferative and clonogenicity potential, expression of stemness genes (*MYC*, *NANOG*, *POU5F1*, and *SOX2*), expression of surface markers (CD73, CD90, CD105, CD14, CD31, CD34, and CD45) and adipo-, chondro- and osteogenic differentiation potential. Then, osteogenic differentiation was induced and the expression of *MSI1* mRNA and other relevant markers of osteogenic differentiation, including *RUNX2* and Periostin, were also evaluated. Results: Cell populations from the alveolar bone (pristine or previously grafted with xenograft), dental follicle, dental germ, dental pulp, and periodontal ligament were obtained. The analysis of proliferative and clonogenicity potential, expression of the stemness genes, expression of surface markers, and differentiation potential showed similar characteristics to those of previously published MSC from the umbilical cord. Under osteogenic differentiation conditions, all MSC populations formed calcium deposits and expressed higher *SPARC*. Over time, the expression of *MSI1* followed different patterns for the different MSC populations. It was not significantly different than the expression of *RUNX2*. In contrast, the expression of *MSI1* and *POSTN* and *RUNX2* were statistically different in most MSC populations. Conclusion: In the current study, a similar expression pattern of *MSI1* and *RUNX2* during in vitro osteogenic differentiation was identified.

## 1. Introduction

Musashi is an RNA-binding protein family formed by two molecules with similar structure (Musashi 1 and Musashi 2). It was described for the first time in 1994 while Nakamura and colleagues were observing sensory organs’ development in drosophila [1]. Both proteins have been principally studied in the nervous system. The genes that encode them are involved in the proliferation of central nervous system stem cells [2].

Musashi 1 (MSI1) binds to its target mRNAs through the cooperative actions of two ribonucleoproteins (RBD1 and RBD2) [3]. MSI1 expression has been detected in stem cells and progenitor cells in the nervous tissue, and at lower levels in differentiated neurons. The molecule binds to mRNA fragments of inductors of cell differentiation, inhibiting their translation. Therefore, MSI1 interferes with cell differentiation and in that way, favors stem/progenitor cells proliferation [4].

One of the most studied target genes of MSI1 is *m-numb*. This gene inhibits the *Notch* pathway, which is involved in the self-renewal of neuronal stem cell. *Notch* is inhibited by *m-numb*, that decreases cell proliferation and enhances cell differentiation. As *m-numb* is silenced by MSI1, the *Notch* pathway continues to maintain the neuronal stem cell self-renewal state [5,6].

Recently, expression of MSI1 has been reported in other tissues, such as eyes, gut, mammary glands, hair follicles, and tissue from the stomach [4]. Our group has also initiated efforts to investigate the potential role of the molecule in bone, which is currently limited. So far, we have identified a histological co-localization of MSI1 with other important regulators of bone metabolism: RUNX2 and Periostin [7]. Thus, in our continuous efforts to better understand the regulator mechanisms of bone regeneration, we aimed to characterize the *MSI1* gene expression along the osteogenic differentiation process of human mesenchymal stromal cells (MSC) in comparison with *RUNX2*, which is a transcription factor known for its implication on osteoblast differentiation [8].

## 2. Results

### 2.1. MSC Characterization

For this study, we first isolated and characterized MSC from distinct intraoral origins, including alveolar bone (ABSC), dental follicle (DFSC), dental germ (DGSC), dental pulp (DPSC), grafted bone (GBSC), and periodontal ligament (PDLSC) samples.

**Figure 1:**
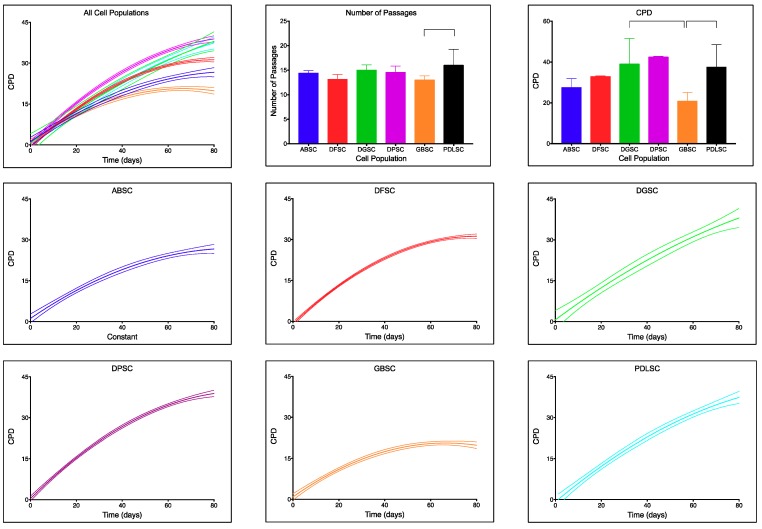
Proliferative curves (dashed lines represent 95% CI), number of passages (mean(SD)) and total accumulated population duplications (CPDs) (mean(SD)) for each cell population. Note the different slopes of each curve and different maximum CPD after 80 days in culture. Only the comparisons between total number of passages for grafted bone (GBSC) and periodontal ligament (PDLSC) (*p* = 0.027; Dunn’s multiple comparison test) and total CPD between GBSC and dental germ (DGSC) (*p* = 0.005; Dunn’s multiple comparison test) and between GBSC and PDLSC (*p* = 0.001; Dunn’s multiple comparison test) were statistically significant.

Each of the cell populations was maintained in culture for up to 80 days. They followed non-linear proliferative rates (Figure 1). Each population followed a different accumulated population duplication (CPD) curve (Table 1) and we were not able to design one curve to statistically fit all populations. In order from lower to higher proliferative potential, GBSC (20.85 ± 4.24), ABSC (27.50 ± 4.36), DFSC (32.86 ± 0.35), PDLSC (37.52 ± 10.94), DGSC (39.04 ± 12.48), and DPSC (42.45 ± 0.34). The final CPD was not statistically significant between the different populations except for the comparison between GBSC and DGSC (*p* = 0.005; Dunn’s multiple comparison test) and between GBSC and PDLSC (*p* = 0.001; Dunn’s multiple comparison test). In all cases, the number of passages was below 20 (GBSC [13.00 ± 0.85], DFSC [13.13 ± 0.99], ABSC [14.40 ± 0.52], DPSC [14.55 ± 1.29], DGSC [15.00 ± 1.10], and PDLSC [16.00 ± 3.27]). Similar to CPD, the number of passages necessary during those 80 days was not statistically significant between the different populations except for the comparison between GBSC and PDLSC (*p* = 0.027; Dunn’s multiple comparison test). Senescence was first detected in passages over 25.

**Figure 2:**
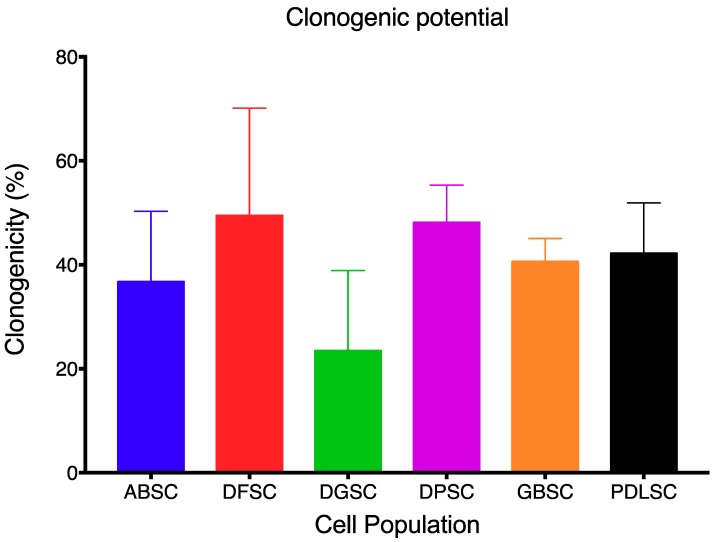
Clonogenicity potential of each cell population. No statistically significant differences were detected. Data are shown as mean (SD).

Each MSC population showed a different clonogenic potential, although comparisons were not statistically significant (*p* = 0.325; Kruskal–Wallis test). From lower to higher clonogenicity potential: DGSC (23.44 ± 15.47), ABSC (36.72 ± 13.59), GBSC (40.63 ± 4.42), PDLSC (42.19 ± 9.72), DPSC (48.13 ± 7.20), and DFSC (49.48 ± 20.64) (Figure 2).

In terms of phenotype, all cell populations showed high positivity for the surface markers CD73, CD90, and CD105 while the expression of CD14, CD31, CD34, and CD45 was negative, as shown in Table 2 and Supplementary Figures S1–S6.

**Figure 3:**
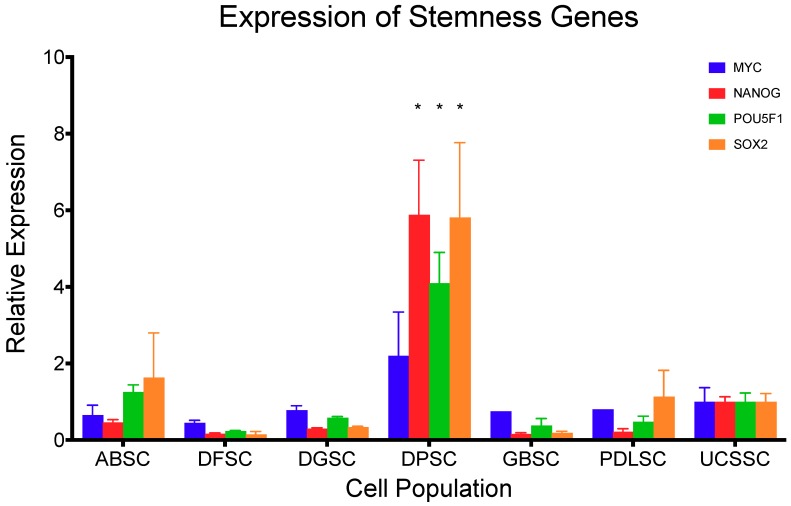
Normalized expression of stemness genes *MYC, NANOG, POU5F1,* and *SOX2* in comparison to mesenchymal stromal cells from the umbilical cord (UCSSC) [9]. Data are shown as mean (SD). *=significant difference compared to UCSSC.

The expression of the stemness gene *MYC* in comparison with the previously established MSC from the umbilical cord (UCSSC) [9] (Figure 3) showed no statistical differences (*p* > 0.213; Dunnett’s multiple comparison test). In contrast, the expression of *NANOG, POU5F1,* and *SOX2* were statistically higher in DPSC (*p* < 0.001; Dunnett’s multiple comparison test) compared to UCSSC, but lower in all of the other cell populations, although not significantly.

**Figure 4:**
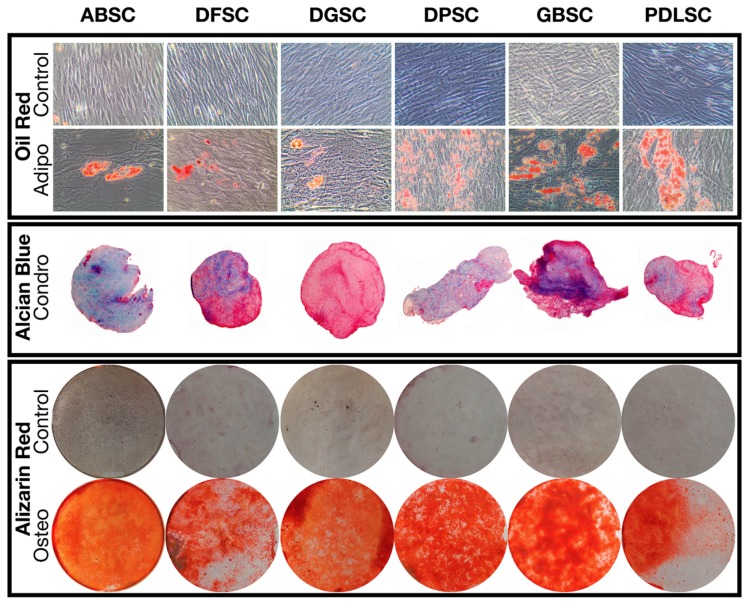
Trilineage differentiation of the different cell populations demonstrating the presence of oil accumulations (oil red staining), sulfated glycosaminoglycans (alcian blue staining), and calcium deposits (alizarin red staining).

**Figure 5:**
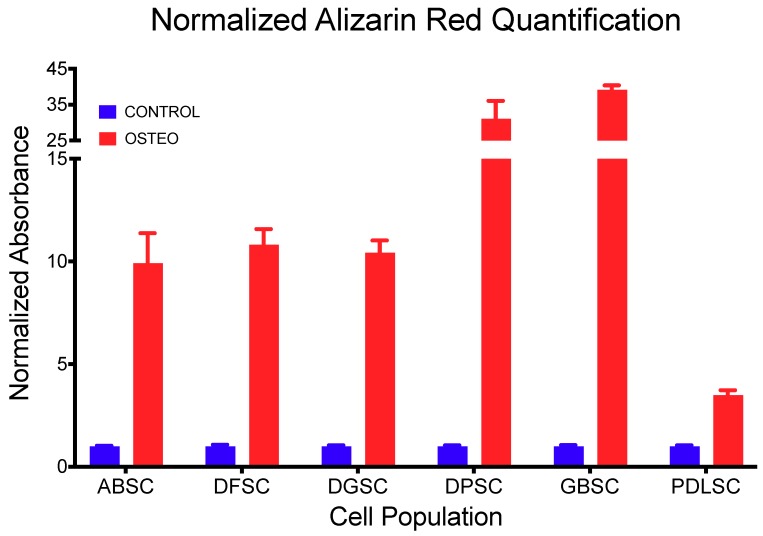
Quantification of alizarin red staining (normalized to control) demonstrating a higher and significant detection in cultures induced to osteoblastic differentiation compared to controls (*p* < 0.001; t test). Data are shown as mean (SD).

The induction of trilineage differentiation into adipocytes, osteoblasts, and chondroblasts of all MSC populations resulted in the formation and detection of oil deposits, sulfated glycosaminoglycans, and calcium deposits, respectively (Figure 4). Particularly, the quantification of alizarin red staining (Table 3 and Figure 5) demonstrated a higher significant detection in cultures induced to osteoblastic differentiation compared to controls (*p* < 0.001; *t*-test).

**Figure 6:**
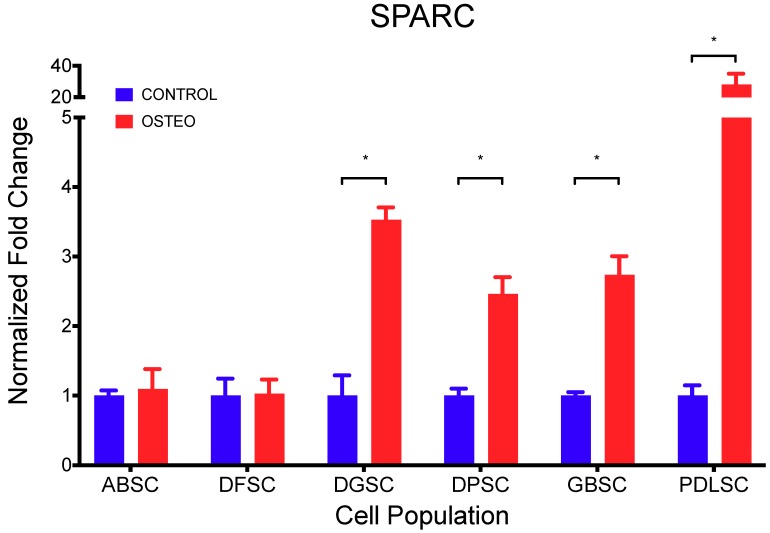
Expression of *SPARC* (fold change normalized to control) demonstrating a higher and significant detection in cultures induced to osteoblastic differentiation compared to controls (*p* < 0.001; *t*-test), except in ABSC (*p* = 0.681) and DFSC (*p* = 0.906). Data are shown as mean (SD).

In addition, the expression of *SPARC* (Table 4 and Figure 6) was significantly higher in cultures induced to osteoblastic differentiation compared to controls (*p* < 0.001; *t*-test), except in ABSC (*p* = 0.681) and DFSC (*p* = 0.906).

### 2.2. Co-Expression of MSI1, RUNX2, and POSTN During Osteogenic Differentiation

**Figure 7:**
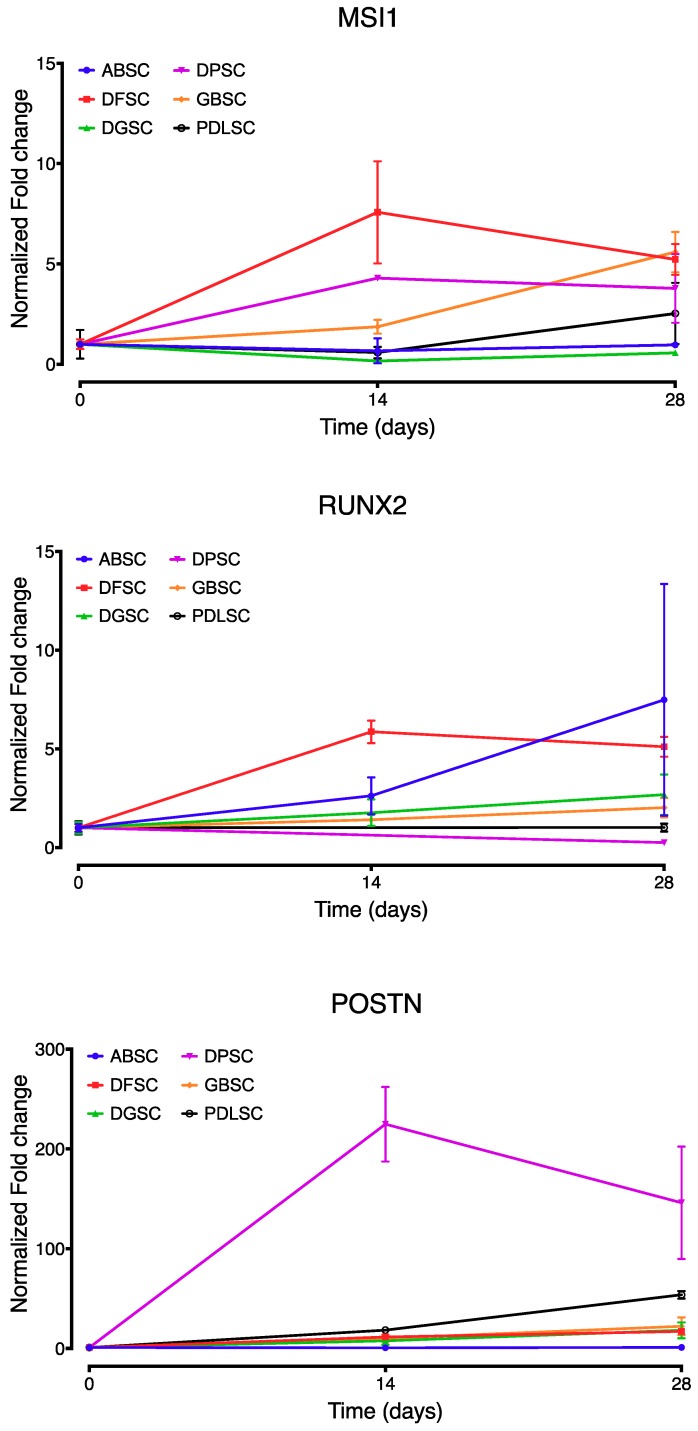
Comparative expression (fold change normalized to day 0) of *MSI1, RUNX2,* and *POSTN* over time by all cell populations under osteogenic conditions. Data are shown as mean (SD).

In osteogenic conditions, as demonstrated by the previous alizarin red and *SPARC* detection, the expression of *MSI1, RUNX2,* and *POSTN* showed comparable patterns between the different cell populations but with isolated differences (Figure 7). At 14 days, *MSI1* was significantly higher in DFSC (*p* < 0.02) compared with the other cell lines, while it was lower in ABSC, DGSC, and PDLSC compared to DPSC (*p* = 0.009, *p* = 0.003 and *p* = 0.007, respectively; Tukey’s multiple comparisons test). At 28 days, *MSI1* was higher in DFSC and GBSC than in ABSC (*p* = 0.002 and *p* = 0.001, respectively; Tukey’s multiple comparisons test), higher in DFSC, DPSC, and GBSC than in DGSC (*p* = 0.001, *p* = 0.022 and *p* < 0.001, respectively; Tukey’s multiple comparisons test) and higher in GBSC than in PDLSC (*p* = 0.032; Tukey’s multiple comparisons test). The expression of POSTN was significantly higher at both 14 and 28 days in DPSC (*p* < 0.001, Tukey’s multiple comparisons test) while it was similar in all other cell populations. The expression of *RUNX2* was not statistically different in any of the cell populations at any time point.

**Figure 8:**
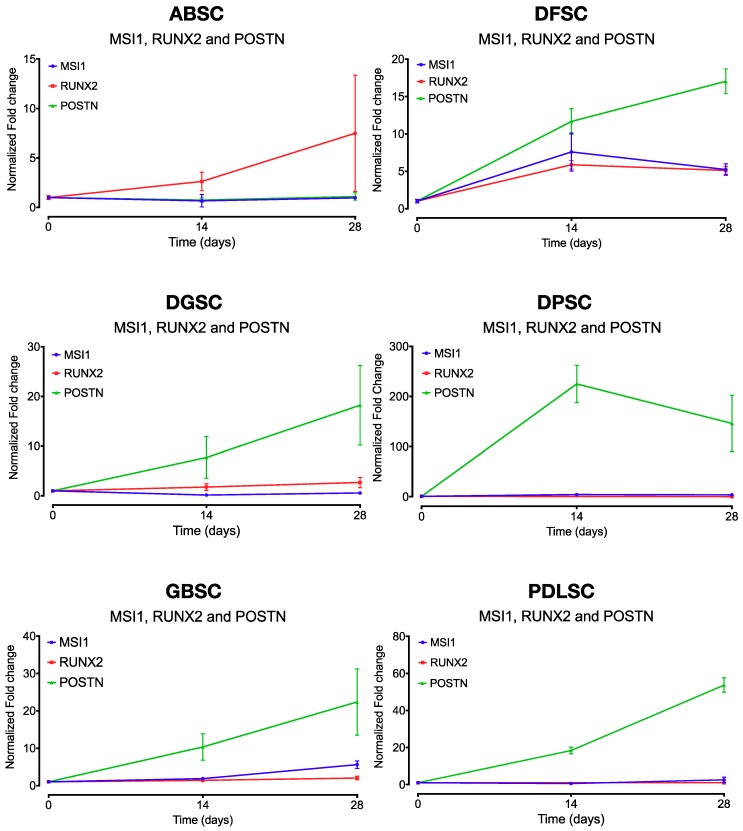
Comparative expression (fold change normalized to day 0) of *MSI1, RUNX2,* and *POSTN* over time by each cell population under osteogenic conditions. Data are shown as mean (SD).

When comparing the relative gene expression of *MSI1, RUNX2,* and *POSTN* within each cell population, there was a statistically significant differential expression of *MSI1* and *RUNX2* only in ABSC (28 days; *p* = 0.024). In all other cell populations, *MSI1* and *RUNX2* followed a comparable pattern of expression under osteogenic differentiation conditions with no significant differences (Figure 8). In contrast, *MSI1* and *POSTN* were statistically different in DFSC (14 and 28 days; *p* = 0.021 and *p* < 0.001, respectively), DGSC (28 days; *p* = 0.001), DPSC (14 and 28 days; *p* = 0.001 and *p* = 0.005, respectively), GBSC (28 days; *p* = 0.001), and PDLSC (14 and 28 days; *p* < 0.001 and *p* < 0.001, respectively). Similarly, *POSTN* and *RUNX2* were statistically different in ABSC (28 days; *p* = 0.027), DFSC (14 and 28 days; *p* = 0.003 and *p* < 0.001, respectively), DGSC (28 days; *p* = 0.002), DPSC (28 days; *p* = 0.004), GBSC (28 days; *p* < 0.001), and PDLSC (28 days; *p* < 0.001). All *p* values above represent Tukey’s multiple comparisons test.

## 3. Discussion

In the particular case of stem cells from intraoral origins, since the initial studies by the group of Gronthos on the multipotency capacities of cells from the dental pulp [10], the exfoliated deciduous tooth [11] and the periodontal ligament [12], many procedures for obtaining, modifying and applying these cells have been proposed [13,14,15]. In the current study, we have characterized stromal cells from most intraoral origins and demonstrated all the properties that a cell population must show to classify it as an undifferentiated multipotent cell. Our results correspond to those previously described in the literature with one particularity: We have applied exactly the same methodology and culture media in all cases. As described, differences were observed in growth kinetics, CPDs, and total number of passages. This reflects the different potentials of each origin which, in fact, is the demonstration of the different embryonic origin and maturation stage of each tissue, as discussed years ago by Sonoyama [16]. For example, it has been demonstrated that intraoral stromal cells from the neural crest (dental pulp stem cells, apical papilla stem cells, and periodontal ligament stem cells, all from the same patient) proliferate at similar rates and exhibit similar clonogenicity activity, in terms of CPD and CFU, than those from alveolar bone [17]. Differentiation potential has also been described to be different [16]. In this manuscript, we have presented a full description of the characterization of each cell origin with the same methodology to establish a foundation for future and deeper studies. Donors of each cell population were different. This limitation may also have influenced the differences in some of the characteristics that have been described.

This study aimed to characterize the expression of *MSI1* during osteogenic differentiation using primary cultures of mesenchymal stromal cells isolated from various intraoral origins. The role of MSI1 on stem cells might be key in the understanding of the healing processes. To know how this protein acts may be important in the development of personalized medicine and regenerative techniques [18]. However, its implications on the osseous tissue remain almost unknown.

MSI1 interferes with cell differentiation and in that way, favors stem/progenitor cells’ proliferation [4]. In fact, the expression of MSI1 influences cell proliferation by targeting c-Myc, p21^CIP1,WAF1^ [19] and p27 [20]. In contrast, it has been assumed that as cells differentiate, MSI1 expression would decrease [21]. For example, our group has identified a decrease in MSI1 expression with osteogenic differentiation when stimulated by Simvastatin on osteoblast-like cells [22]. It must be mentioned, however, that some of these studies were not conducted on primary undifferentiated cells but on immortalized tumor-derived cell lines, such as MG-63. Moreover, some of the currently available literature on this topic reflects the role of MSI1 in neural differentiation but not on osteogenic maturation.

In the current study, we found no differences in the expression of *MSI1* and *RUNX2*, a transcription factor known to regulate osteogenic differentiation [23]. Similar results have been found when we analyzed the expression of MSI1 and RUNX2 in bone regeneration after maxillary sinus floor elevation in human patients [7]. In that study, a high expression of MSI1 was detected in bone-related cells, including MSCs in the non-mineralized bone tissue and osteocytes, osteoblasts, and osteoclasts. Additional differences were also demonstrated when comparing the expression of MSI1 in regenerated and non-regenerated bone, which demonstrates the role of MSI1 in the osteogenic process. This is explained by assuming that the grafted area after 6 months of healing is still active in responding to the grafting material while the native non-grafted area is in a mature quiescent stage. Similar results have been found when using different biomaterials not derived from animals but of synthetic origin (ß-TCP) [24] or modified by adding PLGA coatings to a biphasic calcium phosphate [25]. Similarly, in a different study comparing two distinct biomaterials, we identified a higher expression of MSI1 in biopsies taken from bone grafts that seemed to exhibit an earlier stage of maturation in terms of tissue remodeling activity than in the biopsies with a more advanced maturation [26]. This is, there was a correlation between the expression of MSI1 and a higher vascularization, cellularity, and osteoid lines. Moreover, it was also identified that a biomaterial more colonized by Musashi-1-positive osteoblast precursors was penetrated by more CD34-positive vascular structures [25]. Thus, it could be said that MSI1 is more active when osteogenesis is in process.

In contrast, we have identified significant differences in the expression of *POSTN* compared to the expression of *RUNX2* and *MSI1* during the osteogenic differentiation. In all cases, *POSTN* expression was higher, except when *RUNX2* and *MSI1* were similar, such as in the case of ABSC. POSTN plays an important role in the healing process both in soft [27] and hard tissue [28,29,30]. However, our clinical data do not reflect these differences neither in grafted bone nor in native bone [7]. At this point, we may only elucubrate that since the main role of Periostin is to stabilize the extracellular matrix by increasing collagen fibrillogenesis [31], which would influence osteogenesis by a different path than that in place with the interaction between MSI1 and RUNX2. Deeper studies will be conducted to elucidate such paths.

In summary, our in vitro results of the expression pattern of *MSI1* show their similarity with those of *RUNX2* but differences with *POSTN*. Although they contradict some of the previously published studies on similar topics, those studies did not analyze the osteogenic differentiation in terms of the expression of osteogenic genes, but mostly on neural differentiation or using not-undifferentiated multipotent stromal cells. Furthermore, our previous clinical results demonstrate that MSI1 can be correlated with a higher in vivo osteogenic activity, following similar patterns as those presented here.

## 4. Material and Methods

### 4.1. MSC Collection and Isolation

Mesenchymal stem cells were obtained by explant technique from seven different oral tissues. Alveolar bone (ABSC), dental follicle (DFSC), dental germ (DGSC), dental pulp (DPSC), grafted bone (GBSC), and periodontal ligament (PDLSC) samples were taken from teeth extracted for orthodontic reasons. ABSC and GBSC were obtained from bone during implant placement. Before any collection of the samples, the subjects were informed of the purpose and details of the research, and a written consent of each patient was obtained before any procedure was initiated. The study was conducted under the supervision and after the approval of the Ethics Committee on Human Research from the University of Granada (approval number 424/CEIH/2018).

Immediately after collection of the tissues at the dental clinics of the School of Dentistry—University of Granada, they were submerged in 1X PBS containing 100 U/mL of penicillin/streptomycin (Gibco, Thermo Fisher Scientific, Madrid, Spain) and 0.25 µg/mL of amphotericin B (Sigma-Aldrich, Madrid, Spain), and transferred to the Laboratory of Oral Biology and Regeneration at the Center for Biomedical Research of the University of Granada (CIBM-UGR). The tissues were fragmented with a scalpel and distributed in 6-well plates. Regular Dulbecco’s modified Eagle medium (DMEM) with 1 g/L glucose (DMEM-LG) (Gibco), 10% fetal bovine serum (FBS) (Sigma-Aldrich), 1:100 of non-essential amino acid solution (NEAA) (Gibco), 0.01 μg/mL of basic fibroblast growth factor (bFGF) (PeproTech, London, UK), 100 U/mL of penicillin/streptomycin and 0.25 µg/mL of amphotericin B was added to the culture wells. The cultures were then incubated for 5 days without disturbance and media was then changed every 2 days until cells started to be seen migrating out of the pieces of tissue. After approximately 15 to 20 days, the cells were detached with a solution of 0.08% trypsin-EDTA (Gibco) and subcultured for further experiments (all of them conducted between 4th and 6th passage) or frozen to a stock.

### 4.2. Population Doubling, Growth Kinetic and Senescence

The number of population doublings (PDL) was calculated for each pass by the formula $PDL=\ln2/\ln\;\left(N_{f}⁄N_{i}\right)$, where N_f_ is the number of cells in subconfluence and N_i_ is the initial number of cells seeded. The accumulated population duplication (CPD) up to 80 days has been calculated as the intrinsic value of the age of a cell population in culture. CPD indicates the total number of times that a population has been able to duplicate from its isolation as a function of time in days of cultivation.

The CPD for each cell population has been represented in graphs. A quadratic non-linear curve has been fitted to each data set. From there, the R^2^ (goodness of fit between the curve and the data) and the formula for the model have been obtained. By doing that, we can use the formula in future experiments to predict the cell growth rate. In addition, the extra sum-of-squares F test has been used to determine comparability between growing curves among cell populations.

To compare among the different origins, we have compared the curves with the extra sum-of-squares F test and setting the statistical significance at a *p*-value of less than 0.05.

To evaluate the senescence of each cell population, a β-galactosidase staining analysis was conducted after the last passage of the cells used for analyzing the CPD. A Senescence β-Galactosidase Staining Kit (Cell Signaling Technology, Frankfurt, Germany) was used following the manufacturer instructions.

### 4.3. Clonogenicity Potential

To analyze the clonogenicity potential or self-renewing capacities of the stem cells, we studied the capacity to form colonies from single cells (colony forming units, CFU). To do so, we conducted the experiments by a limiting dilution technique. A cell suspension containing a total of 32 cells was seeded in a 96 multi-well plate. By doing so, the probability to seed a cell alone each 3 wells is maximized.

After 14 days in culture with the regular media described above, all the wells were treated with ethanol 70% to fix the culture. Then, crystal violet (methyl violet 10B or hexamethyl pararosaniline chloride) was added to stain the cells wherever they were present. Then, the number of wells with at least 50 cells were recorded. This informs of the fact that the cell that was originally in that well had been able to form a colony, i.e., it was able to self-renew.

To calculate the clonogenicity potential, the following formula was used:

$$\frac{Number\;of\;wells\;with\;colonies\;after\;14\;days\;\times \;100}{Total\;number\;of\;cells\;seeded}$$

For each cell population, the mean (standard deviation) was calculated. Because all the data were normally distributed (Shapiro–Wilk normality test) and the measures were independent, differences between the cell populations were calculated with a one-way ANOVA test. To evaluate pair-wise comparisons, Bonferroni’s multiple comparisons post-hoc test was used with a *p*-value of less than 0.05 as statistically significant.

### 4.4. Phenotype

An analysis of the expression of characteristic surface markers was conducted by flow cytometry using the FACSCanto II cytometer (BD Biosciences, Madrid, Spain) on 10^5^ cells and the manufacturer recommended concentration of each antibody. Anti-CD90 conjugated with fluorescein-5-isothiocyanate (FITC) (eBioscence, clone eBio5E10), anti-CD73 conjugated with phycoerythrin (PE) (BD Pharmingen, cloneAD2-PE) and anti-CD105 conjugated with allophycocyanin (APC) (Serotec, clone SN6) were studied for positivity; anti-CD14-FITC (BD, clone M5E2), anti-CD31-PE (R&D, clone 9G11), anti-CD34-PE (BD, clone 2D1) and anti-CD45-APC (Caltag, clone HI30) were evaluated for negativity. The corresponding isotype controls were also evaluated.

### 4.5. Stemness

Stemness was evaluated by analyzing the expression of *MYC, NANOG, POU5F1,* and *SOX2* in comparison to the expression of these genes in an umbilical cord-derived stem cell population previously evaluated by our group [9]. Briefly, cells were cultured in standard conditions until 80%–90% confluency in 6-well plates. Then, after washing 2 times with 1XPBS, 1 mL of TRIzol^®^ was added, collected and frozen at −80 °C until RNA extraction. To do so, 200 µL of chloroform was added, the samples vortexed for 15 s, and incubated at room temperature for 3 min. Then, the tube was spun at 12,000 × g for 10 min at 4 °C. After that, 350 µL of the aqueous phase was obtained and 0.5 µL of glycogen added to precipitate the RNA, to which 500 µL of 100% isopropanol was added, incubated for 10 min, and centrifuged again at 12,000 × g for 10 min at 4 °C. The pellet was washed with 1 mL of 75% ethanol, vortexed, centrifuged at 7500 × g for 5 min at 4 °C and, finally, when the pellet had dried, 20 µL of RNAsa free water was added to obtain a final solution that was incubated for 15 min at 60 °C. The RNA concentration was then measured using a NanoDrop. cDNA was obtained by preparing 10 µL of 1X PrimeScript RT Master Mix (Takara, Saint-Germain-en-Laye, France), 500 ng of RNA and RNAsa free water. The reaction was made in a Procoge thermocycler (Techne, Staffordshire, UK) for 15 min at 37 °C followed by 5 s at 85 °C. Twenty microliters of EASY dilution was then added for a final volume of 30 µL of cDNA.

With a concentration of 5 µM of the primers listed in Table 5, the qPCR was conducted using 1X iQ SYBR^®^ Green Supermix, 2 µl of cDNA and RNAsa free water for a final volume of 20 µL using the iCycler (Bio-Rad). An initial activation phase of 2 min at 95 °C was followed by 30 cycles of 15 s at 94 °C, 30 s at 60 °C, and 30 s at 72 °C. Relative expression of each gene was calculated with the 2^−ΔΔ*C*t^ method normalized to *GAPDH*. An additional housekeeping gene (*RPLP0*) was used as confirmation of the results. In this case, for comparative purposes, a second normalization was made to the expression of the genes in the umbilical cord-derived population.

### 4.6. Trilineage Differentiation Potential

To further characterize the stemness of the different populations, trilineage differentiation was induced and evaluated as follows.

#### 4.6.1. Adipogenic Differentiation

To induce cells to adipogenic differentiation, cells were seeded at 4000 cells/cm^2^ in 12-well plates and regular media was added supplemented with 1% insulin–transferrin–selenium (41400045, Invitrogen, Thermo Fisher Scientific), 1 µM of dexamethasone (Sigma-Aldrich, D2915), 0.2 mM of indomethacin (Sigma-Aldrich, I7378), and 1 µM of isobutylmethylxanthine (IBMX) (Sigma-Aldrich, I7018). The culture was maintained for 28 days with media changed every 3 days. Then, the cells were fixed with 10% formalin for 1 h at room temperature, washed with 60% isopropanol and dried completely before adding the oil red working solution (300 mg of oil red in 100 mL isopropanol 99% in a 3:2 proportion with ddH2O) for 10 min. The solution was then removed, and the wells immediately washed four times with dH_2_O. Pictures were taken at this point.

#### 4.6.2. Chondrogenic Differentiation

Chondrogenic differentiation was induced to cell pellets seeded in a U-shaped-bottom 96-well plate at a density of 200,000 cells per well. The cells were then incubated for 48 h until a spheroid was formed. Then, media was changed to Mesenchymal Stem Cell Chondrogenic Different Medium (C28012, Promocell, Heidelberg, Germany) and changed again every 3 days until day 21. After 21 days, the media was removed carefully and the spheroids fixed with 4% paraformaldehyde (PFA) for 20 min at room temperature. Fixed spheroids were then washed twice in 1X PBS, transferred to 70% ethanol and stored at 4 ºC until inclusion in paraffin and sectioned for standard alcian blue staining. Evaluation of chondrogenic differentiation was made by a 0–3 categorical scale (0 = no differentiation; 3 = maximum differentiation).

#### 4.6.3. Osteogenic Differentiation

Osteogenic differentiation was evaluated by alizarin red staining and analysis of the expression of *RUNX2* and *SPARC* genes after inducing their differentiation. For osteogenic differentiation, 3000 cells/cm^2^ were seeded onto 6-well plates for RNA evaluation and 12-well plates for alizarin red staining. Osteogenic media consisted of regular media as described above supplemented with 10 mM of β-glycerophosphate (50020, Fluka, Bucharest, Romania), 0.1 µM of dexamethasone (D2915, Sigma-Aldrich), and 0.05 mM of L-ascorbic acid (A8960, Sigma-Aldrich). Cultures were maintained for 28 days with media changed every 3 days. Then, RNA was collected and processed for the evaluation of osteogenic markers as described above. The other groups of samples were stained for alizarin red. Briefly, media was removed from the wells, cells were then washed twice with 1X PBS and fixed with 4% PFA for 15 min at room temperature. Cells were then washed twice with ddH_2_O and stained with a working solution of 40 mM alizarin red at pH 4.2 for 20 min at room temperature and gentle shaking. Then, the excess of stain was removed by washing four times with ddH_2_O. The wells were dried at room temperature for 1 week and photos captured. For the quantification of alizarin red staining, stained cells were incubated with 1 mL of a solution of 10% cetylpyridinium chloride under shaking conditions for 1 h. The dye was then removed, and 200 µL aliquots were transferred to a 96-well plate. The level of calcium from the cultured cells was detected by measuring absorbance at 550 nm using a microplate reader.

### 4.7. Temporal Expression of Musashi-1

For the evaluation of Musashi-1 (*MSI1*) during osteogenic differentiation, RNA was collected at time 0 days (T0), T14, and T28. qPCR was conducted as described above. Additionally, to contextualize the expression of this marker, comparisons between the expression of the osteogenic marker *RUNX2* and *MSI1* as well as between *POSTN* and *MSI1* were conducted.

## 5. Conclusions

The understanding of bone physiology along the healing processes is a key factor to improve bone regeneration. The role of Musashi-1 in those events seems to be of great importance, although it has hardly been studied. All the acquired knowledge in this line of research could be very useful to implement cell and gene therapy in bone regeneration.

In the current study, we have identified a similar expression pattern of *MSI1* and *RUNX2* during in vitro osteogenic differentiation.

## Figures and Tables

**Table 1:** Best-fit values of accumulated population duplications (CPD) curves for each cell population.

	ABSC	DFSC	DGSC	DPSC	GBSC	PDLSC
Best-fit values						
B0	1.142	−0.8773	0.6326	0.3618	0.8496	−0.8511
B1	0.5771	0.7993	0.6278	0.837	0.5941	0.7096
B2	−0.003228	−0.004974	−0.002007	−0.004444	−0.00446	−0.002893
CPD	27.50 ± 4.36	32.86 ± 0.35	39.04 ± 12.48	42.45 ± 0.34	20.85 ± 4.24	37.52 ± 10.94
Number of passages	14.40 ± 0.52	13.13 ± 0.99	15.00 ± 1.10	14.55 ± 1.29	13.00 ± 0.85	16.00 ± 3.27

**Table 2:** Expression of mesenchymal stromal cell (MSC) surface markers.

**ABSC**	**% Alive**	82.40%				
	**CD73/CD90/CD105**		99.69%	97.90%		98.80%
	**CD34/CD45/CD14/CD31**		1.79%	1.28%	0.03%	1.31%
**DFSC**	**% Alive**		71.60%			
	**CD73/CD90/CD105**		99.92%	99.16%		97.38%
	**CD34/CD45/CD14/CD31**		0.99%	1.83%	0.02%	0.05%
**DGSC**	**% Alive**		95.40%			
	**CD73/CD90/CD105**		99.90%	84.30%		66.90%
	**CD34/CD45/CD14/CD31**		2.31%	0.31%	0.11%	0.05%
**DPSC**	**% Alive**		75.30%			
	**CD73/CD90/CD105**		99.95%	96.86%		96.75%
	**CD34/CD45/CD14/CD31**		0.88%	1.12%	0.02%	0.06%
**GBSC**	**% Alive**		90.00%			
	**CD73/CD90/CD105**		99.78%	96.37%		98.40%
	**CD34/CD45/CD14/CD31**		0.45%	1.12%	0%	1.05%
**PDLSC**	**% Alive**		88.30%			
	**CD73/CD90/CD105**		99.61%	99.80%		98.69%
	**CD34/CD45/CD14/CD31**		0.43%	0.4%	0%	0.09%

**Table 3:** Normalized detection of Alizarin Red staining after 28 days in osteogenic induction media.

	CONTROL		OSTEO		*p* Value (*T-*Test)
	Mean	SD	Mean	SD	
**ABSC**	1.000	0.027	9.926	1.448	0.001
**DFSC**	1.000	0.069	10.817	0.761	0.000
**DGSC**	1.000	0.050	10.427	0.598	0.000
**DPSC**	1.000	0.049	31.111	4.955	0.001
**GBSC**	1.000	0.060	39.197	1.176	0.000
**PDLSC**	1.000	0.041	3.498	0.227	0.000

**Table 4:** Normalized expression of *SPARC* after 28 days in osteogenic induction media.

	CONTROL		OSTEO		*p* Value (*T*-Test)
	Mean	SD	Mean	SD	
**ABSC**	1.000	0.073	1.098	0.283	0.681
**DFSC**	1.000	0.243	1.030	0.201	0.906
**DGSC**	1.000	0.290	3.531	0.173	0.008
**DPSC**	1.000	0.098	2.462	0.241	0.015
**GBSC**	1.000	0.049	2.737	0.268	0.012
**PDLSC**	1.000	0.147	28.125	6.824	0.030

**Table 5:** Sequence of each of the primers for the genes evaluated in the current study.

Gene	Primer Forward	Primer Reverse
*MYC*	GGAGATCCGGAGCGAATAGGG	GTTTCGTGGATGCGGCAAGG
*NANOG*	TGAATCCTTCCTCTCCCCTCC	CCTCGCTGATTAGGCTCCAAC
*POU5F1*	AGGCCCGAAAGAGAAAGCGA	CTGATCTGCTGCAGTGTGGGT
*SOX2*	TCAGGAGTTGTCAAGGCAGAG	CCGCCGCCGATGATTGTT
*MSI1*	TGAGCAGTTTGGGAAGGTG	TCACACACTTTCTCCACGATG
*POSTN*	TTTCTACTGGAGGTGGAGAAAC	GTGACCTTGGTGACCTCTTC
*SPARC*	AGAACAACACCCCCATGTGCGT	TCCAGGGTGCACTTTGTGGCAA
*RUNX2*	ACCGTCTTCACAAATCCTCCC	AGCTTCTGTCTGTGCCTTCTG
*GAPDH*	AGCTCATTTCCTGGTATGACAAC	TTACTCCTTGGAGGCCATGTG
*RPLP0*	CAGATTGGCTACCCAACTGTT	GGCCAGGACTCGTTTGTACC

## Supplementary Materials

Supplementary materials can be found at https://www.mdpi.com/1422-0067/20/9/2171/s1.
